# Trends in health reforms in the Health System and Policies Monitor network: increasing interaction of primary health care and care coordination

**DOI:** 10.1093/eurpub/ckac129.624

**Published:** 2022-10-25

**Authors:** K Polin, W Quentin

**Affiliations:** 1 Department of Health Care Management, Technical University Berlin, Berlin, Germany; 2 European Observatory on Health Systems and Policies, Technical University Berlin, Berlin, Germany

## Abstract

Since 2018, the Health System and Policy Monitor (HSPM) network of the European Observatory on Health Systems and Policies has tracked health reform activity in its countries. Top three reforms are identified by national experts yearly and classified across 11 policy clusters. Efforts inform a standardized overview of healthcare development. 337 reforms have been assessed for 31 (mainly EU) countries over 4 years, with clear trends like the converging of PHC and care coordination. Main reform areas were primary/ambulatory care, governance, and hospital care (2018-2019); coordination, governance, and digital health (2020); and care coordination, governance, and insurance coverage/resource generation (2021). For PHC, this suggests a shift from leveraging innovations to support existing delivery mechanisms to transforming PHC. Some PHC reforms in 2018-2019 introduced elements of integrated care, but more focused narrowly on specific levers, e.g., gatekeeping. Early care coordination reforms focused on certain patient groups or care delivery across the system. Separately, PHC and care coordination have long been reform priorities. Since 2020, they are increasingly linked to one another, and to multidisciplinary care. This suggests a recognition that multidisciplinary processes/staffing are key to realizing the promises of PHC-to coordinate the care of patients and ensure comprehensive and continuous care over time for most health needs. This vision is underpinned by the UN SDGs and COVID-19, which highlighted the need for health systems to be set up for and with the resources to respond to crises while maintaining core functions. The overview of HSPM health reforms identifies reform trends and opportunities for cross-country learning. More research is needed for policymakers to benefit fully from reform experiences abroad. Case studies with in-depth analysis of reform design and implementation processes can help inspire or support future health reforms elsewhere.

